# Community participation, physical activity, and quality of life for children born very preterm

**DOI:** 10.1111/dmcn.16295

**Published:** 2025-03-20

**Authors:** Kate L. Cameron, Rheanna M. Mainzer, Joy E. Olsen, Ross A. Clark, Jeanie L. Y. Cheong, Tara L. FitzGerald, Alicia J. Spittle

**Affiliations:** ^1^ Victorian Infant Brain Studies, Clinical Sciences Murdoch Children’s Research Institute Melbourne VIC Australia; ^2^ Department of Physiotherapy University of Melbourne Melbourne VIC Australia; ^3^ Department of Paediatrics University of Melbourne Melbourne VIC Australia; ^4^ Clinical Epidemiology and Biostatistics Unit Murdoch Children’s Research Institute Melbourne VIC Australia; ^5^ Neonatal Research The Royal Women’s Hospital Melbourne VIC Australia; ^6^ School of Health University of the Sunshine Coast Queensland VIC Australia; ^7^ Department of Obstetrics and Gynaecology University of Melbourne Melbourne VIC Australia

## Abstract

**Aim:**

To investigate the effects of community environment on physical activity and quality of life (QoL) and to describe the relationship between community participation with physical activity and QoL, in children born very preterm and at term.

**Method:**

Participants in this cross‐sectional study were 45 children aged 4 to 5 years old, born before 30 weeks' gestation and 89 term‐born children. Measures were community participation (Young Children's Participation and Environment Measure; frequency, involvement, environmental helpfulness, environmental resources), QoL (Pediatric Quality of Life Inventory 4.0; PedsQL), and physical activity (7‐day accelerometry). Effects of environmental helpfulness and resources on physical activity and QoL were estimated using g‐computation. Relationships between participation frequency and involvement with physical activity and QoL were estimated using linear regression.

**Results:**

Environmental helpfulness increased physical activity (average minute increase per percentage point environmental helpfulness score increase; 3, 95% confidence interval [CI] 0.1–7), reduced stationary time (−2, 95% CI −5 to −0.2) and improved QoL (average PedsQL social functioning score increase; 0.7, 95% CI 0.1–1.2). Greater community involvement was associated with better QoL (average PedsQL total score increase per unit involvement score increase; 5.8, 95% CI 0.7–10.9).

**Interpretation:**

Improving environmental helpfulness may improve physical activity and QoL for 4‐ to 5‐year‐old children. Greater community involvement is associated with better QoL.

## Graphical abstract

Overall, we found that more helpful and better resourced community environments improved physical activity and quality of life for 4–5‐year‐old children, while children who were more involved in their community activities tended to have higher quality of life. These findings were similar for children who were born < 30 weeks’ gestation and at term. This paper highlights the potential for interventions which promote community participation or improve community environments when aiming to support physical activity and quality of life for preschool age children.

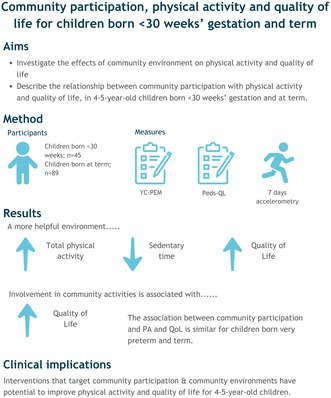

Plain language summary: https://onlinelibrary.wiley.com/doi/10.1111/dmcn.16337

This original article is commented by Killeen on pages 1246–1247 of this issue.

**Abbreviations**
PedsQL: Pediatric Quality of Life Inventory 4.0
QoL: quality of life
VPT: very preterm
YC‐PEM: Young Children's Participation and Environment Measure

**What this paper adds**

- Improving environmental helpfulness could positively impact physical activity and quality of life (QoL) for 4‐ to 5‐year‐old children born preterm and at term.
- Greater involvement in community activities is associated with a higher QoL.
- The association between community participation, physical activity, and QoL is similar for children born very preterm and term.

Children born very preterm (VPT; <32 weeks' gestation) are at greater risk of poor health and developmental outcomes, including motor^1^ and cognitive impairment,^2^ behavioural and social difficulties,^3^ chronic lung disease,^1^ and low bone density,^4^ compared with children born at term. Given these multidimensional comorbidities of VPT births, strategies to support children born VPT to live healthy, active lifestyles are needed. Facilitating participation in community‐based activities (such as sports teams, community events, classes, and family outings) may be a useful way of promoting health and quality of life (QoL) for children born VPT.

The family of Participation‐Related Constructs conceptualizes participation as both attendance (being there) and involvement (the subjective experience of participating) in meaningful life situations.^5^ It also emphasizes that participation occurs in context, and is influenced by the physical and social environment in which participation takes place.^5^ The family of Participation‐Related Constructs emphasizes that participation is an important outcome for individuals, but can also be an intervention itself.^5^ Research into participation for children born VPT is an emerging field. While studies have considered barriers and facilitators to participation,^6^, ^7^ as well as potential correlates of participation (e.g. social risk, motor impairment),^8^, ^9^ there is limited research investigating how community participation may influence aspects of health and well‐being. Understanding how participation may benefit different health domains is essential when facilitating participation as part of an intervention, and in communicating the benefits of participation to children and families.

The aims of this study were to: (1) estimate the effects of helpful environmental features and environmental resources on objectively measured physical activity and QoL for preschool‐age children born before 30 weeks' gestation and at term; and (2) describe the relationship between community participation (attendance and involvement) with physical activity and QoL for preschool‐age children born before 30 weeks' gestation and at term. We additionally estimated these effects and relationships for each birth group. We hypothesized that a more helpful and better resourced environment would have a positive effect on both physical activity and QoL, and that community participation would be positively associated with both physical activity and QoL.

## METHOD

### Participants

Participants were recruited at birth between January 2011 and December 2013 from the Royal Women's Hospital, Melbourne, Australia, as part of a prospective longitudinal cohort study comparing neurobehavioural development of children born before 30 weeks (*n* = 143 survivors) with term‐born individuals (*n* = 151), known as the Victorian Infant Brain Study‐2.^10^ Inclusion and exclusion criteria for this cohort have been described elsewhere.^10^ Children who attended a follow‐up appointment between 4 years and 5 years corrected age^10^ were eligible for the current cross‐sectional analysis of the Victorian Infant Brain Study‐2 cohort. This study was approved by the Human Research Ethics Committee at the Royal Children's Hospital, Melbourne, Australia. Parents gave written informed consent for their child to participate.

### Procedures and outcomes

Perinatal characteristics were collected during the newborn period, while data were collected for the 4‐ to 5‐year follow‐up between April 2016 and January 2019. Questionnaires were completed by the primary caregiver.

#### Community participation

Community participation was assessed using the community section of the Young Children's Participation and Environment Measure (YC‐PEM).^11^ The YC‐PEM is a valid and reliable caregiver‐completed questionnaire for children aged 0 to 5 years with and without developmental delays.^12^ This study uses four scales from the YC‐PEM: environmental helpfulness, environmental resources, frequency of community participation, and involvement in community participation. Environmental helpfulness and environmental resources measure aspects of the child's environment, such as the physical layout of community spaces, policies, public transport, and funding, which are not very susceptible to reverse causality as they are stable over time, occur before physical activity and QoL variables, and are not influenced by physical activity or QoL. Environmental helpfulness is determined by considering the helpfulness of 10 environmental features for facilitating community participation: physical layout, sensory qualities, physical demands, cognitive demands, social demands, attitudes of others, child relationships, weather, safety, and policies. Answers are scored as 3 (usually helps or no impact), 2 (sometimes helps, sometimes makes harder), or 1 (usually makes harder). Scores are summed and expressed as a percentage of each individual's total possible score. To measure the availability/adequacy of environmental resources, seven environmental resources are considered: public transportation, private transportation, programmes in the community, equipment or supplies, information about activities, time, and money. Answers are scored as 3 (usually yes or not needed), 2 (sometimes yes, sometimes no), or 1 (usually no). Again, scores are summed and expressed as a percentage of each individual's total possible score. To measure frequency of participation, how often a child participates in 11 different community activities is scored on an ordinal Likert scale, ranging from 0 (never) to 7 (once or more each day). Scores are summed and divided by the number of activities in which the child participates. For involvement in community participation, how involved a child is in each activity is scored using an ordinal Likert scale from 1 (not very involved) to 5 (very involved). Again, scores are summed and divided by the number of activities in which the child participates.

#### Physical activity

Physical activity was assessed using a triaxial accelerometer (Axivity AX3, Newcastle upon Tyne, UK) which has been used in other studies involving children.^13^, ^14^ The accelerometer was placed inside a band and fitted to each participant's ankle during the assessment. Participants wore the accelerometer for the next 6 consecutive days. The Axivity AX3 accelerometer is water‐resistant, and parents were instructed not to remove it during swimming/bathing where possible.

#### QoL

QoL was measured using the Pediatric Quality of Life Inventory 4.0 (PedsQL), which is a valid and reliable parent‐report measure of child health‐related QoL. The PedsQL measures the core dimensions of health as described by the World Health Organization. It has four scale scores (physical, social, emotional, and school functioning) and three summary scores (physical health and psychosocial health summary scores, and total score), with higher scores indicating better QoL.^15^ We did not include school functioning scores in this study, as most children were not yet attending school.

#### Social risk status

Social risk status was obtained through parental questionnaires using a composite measure^16^ of six elements known to influence child development: family structure; primary caregiver education; primary income earner occupation and employment; language spoken at home; and maternal age at birth. Each item was scored 0, 1, or 2, and summed to give an overall score. Higher social risk was categorized as scores ≥2.

### Data management

Accelerometers were initialized using OmGui software (Open Movement, Newcastle University, Newcastle upon Tyne, UK) and set to record for 7 days from each participant's appointment time. The assessment day was removed from analysis, as it did not capture a full 24 hours, leaving a maximum of 6 complete days of accelerometer data. Information about the analysis program and accelerometer processing guidelines can be found in our previous study.^14^ A valid day of data was defined as at least 10 hours' wear which is consistent with existing recommendations.^17^ Participants with at least 3 valid days were included in the analyses.

Five physical activity outcomes were obtained using different methods to capture the spectrum of daily movement. Total physical activity (minutes/day) was determined by summing each non‐stationary minute during waking hours. A subset of total physical activity was minutes spent stepping per day (stepping physical activity) and total number of steps per day. The number of daily fast steps were also summed and used as a measure of moderate‐vigorous physical activity. To determine fast steps, the individual participant's modal stride rate (i.e. most common stride speed) over the days of accelerometer wear (in hertz) was multiplied by 1.31 to identify a fast‐step threshold for each participant.^18^ We measured stationary behaviour as waking behaviour completed while lying/reclining/sitting/standing without ambulation, regardless of energy expenditure, which is consistent with the Sedentary Behaviour Research Network consensus terminology.^19^ Stationary time (minutes/day) was obtained by summing each minute with no recorded steps during waking hours.

### Statistical analysis

Data were analysed using Stata version 17.0 (StataCorp, College Station, TX, USA). The directed acyclic graph in Figure S1 outlines causal assumptions made in the analysis for aim 1. Covariates used to control for confounding were selected a priori on the basis of clinical knowledge and a review of the literature.^6^, ^9^, ^14^, ^20^, ^21^, ^22^, ^23^, ^24^, ^25^ The causal contrasts of interest were the mean differences in physical activity and QoL outcomes for a one‐unit increase in environmental helpfulness and resources exposures. These contrasts were estimated using g‐computation. Linear regression models were used to predict the outcome under different exposure values. Models included social risk status, birth group, and motor impairment (models involving QoL only) as main effects, and an exposure × birth‐group interaction. Confidence intervals (CIs) were obtained using a non‐parametric cluster bootstrap (percentile method) to account for clustering among multiple births in a family. Since the aim was to estimate the causal effect, results were interpreted focusing on point estimates and CIs.

For aim 2, mean differences in physical activity and QoL outcomes for a one‐unit increase in community participation attendance and involvement were estimated using linear regression models, both with and without including birth group and a participation × birth‐group interaction. Models were fitted using generalized estimating equations with an exchangeable correlation structure to account for clustering among multiple births in a family and using robust (sandwich) standard errors. When models could not be fitted using generalized estimating equations (owing to non‐convergence), they were fitted using ordinary linear regression and reported with robust standard errors that allowed for clustering. Estimates are reported with 95% CIs and *p*‐values, both overall and separately by birth group.

## RESULTS

Participants were included in this study if they had YC‐PEM data in addition to physical activity and/or QoL data. The characteristics of the 134 participants included in this study (*n* = 45 < 30 weeks; *n* = 89 term) are presented in Table 1. The numbers of participants completing each measure and reasons for missing data are outlined in Figure S2. The characteristics of those included in the current study were similar to those who were not included (Table S1), although a greater proportion of included participants born before 30 weeks were twins/triplets than non‐participants born before 30 weeks.

**TABLE 1:** Summaries of participants' characteristics and outcomes (*n* = 134).

Characteristics	Born <30 weeks, *n* = 45	Term‐born, *n* = 89
Gestational age (weeks), mean (SD) (range)	27.7 (1.7) (23.6–29.9)	39.9 (1.2) (37–42)
Corrected age at assessment (years:months), mean (SD) (range)	4:8 (0:1) (4:5–5:0)	4:10 (0:2) (4:5–5:5)
Multiple births, *n* (%)	20 (44)	2 (2)
Sex (male), *n* (%)	21 (47)	44 (49)
Higher social risk, *n* (%)^a^	18/39 (46)^b^	22/85 (26)^b^
MABC‐2 ≤ 16th centile and/or cerebral palsy, *n* (%)	17/44 (39)^b^	12/87 (14)^b^
Cerebral palsy diagnosis, *n* (%)	3 (7)	0 (0)
L‐DCDQ suspect for DCD, *n* (%)	21/41 (51)^b^	29/83 (35)^b^
Autism spectrum disorder diagnosis, *n* (%)	2 (4)	2 (2)
**YC‐PEM outcomes, median (IQR) (range)**		
Community participation frequency	4.1 (3.6–4.4) (2.2–5)	4.3 (3.9–4.7) (2.8–5.3)
Community participation involvement	4.5 (3.8–4.6) (2.6–5)	4.5 (4.1–4.8) (2.0–5.0)
Helpfulness of environmental features	86.7 (80–100) (70–100)	93.3 (90–100) (66.7–100)
Availability environmental resources	76.2 (42.9–90.5) (28.6–100)	90.5 (42.9–95.2) (28.6–100)
**Physical activity outcomes, median (IQR) (range)** c		
Total physical activity (minutes/day)	473 (406–517) (195–659)	519 (465–553) (177–661)
Stepping physical activity (minutes/day)	178 (128–206) (108–261)	189 (163–223) (72–305)
Stationary time (minutes/day)	341 (279–372) (218–432)	301 (273–344) (173–548)
Steps (*n*/day)	12809 (9689–15 323) (7954–19 951)	14110 (12 057–16 760) (4809–21 921)
Fast steps (*n*/day)	4019 (2944–5744) (2008–10 435)	4812 (3887–5582) (2273–9148)
**PedsQL outcomes, median (IQR) (range)** d		
Total score	78.3 (64.1–85.9) (29.3–93.5)	80.4 (72.8–90.2) (38–100)
Physical health score	84.4 (75–93.8) (25–100)	90.6 (81.2–93.8) (12.5–100)
Psychosocial health score	75 (63.3–85.0) (31.7–93.3)	78.3 (63.3–90) (41.7–100)
Emotional functioning score	70 (60–80) (40–100)	75 (65–85) (30–100)
Social functioning score	70 (55–95) (15–100)	80 (60–95) (30–100)

Abbreviations: DCD, developmental coordination disorder; IQR, interquartile range; L‐DCDQ, Little Developmental Coordination Disorder Questionnaire; MABC‐2, Movement Assessment Battery for Children, Second Edition; PedsQL, Pediatric Quality of Life Inventory 4.0; SD, standard deviation; YC‐PEM, Young Children's Participation and Environment Measure.

^a^ Higher social risk defined as social risk index score ≥2.

^b^ *n*/number with available data.

^c^ Data for *n* = 39 very preterm, *n* = 84 term.

^d^ Data for *n* = 41 very preterm, *n* = 87 term.

### Environment (helpfulness and resources) and physical activity

**FIGURE 1:**
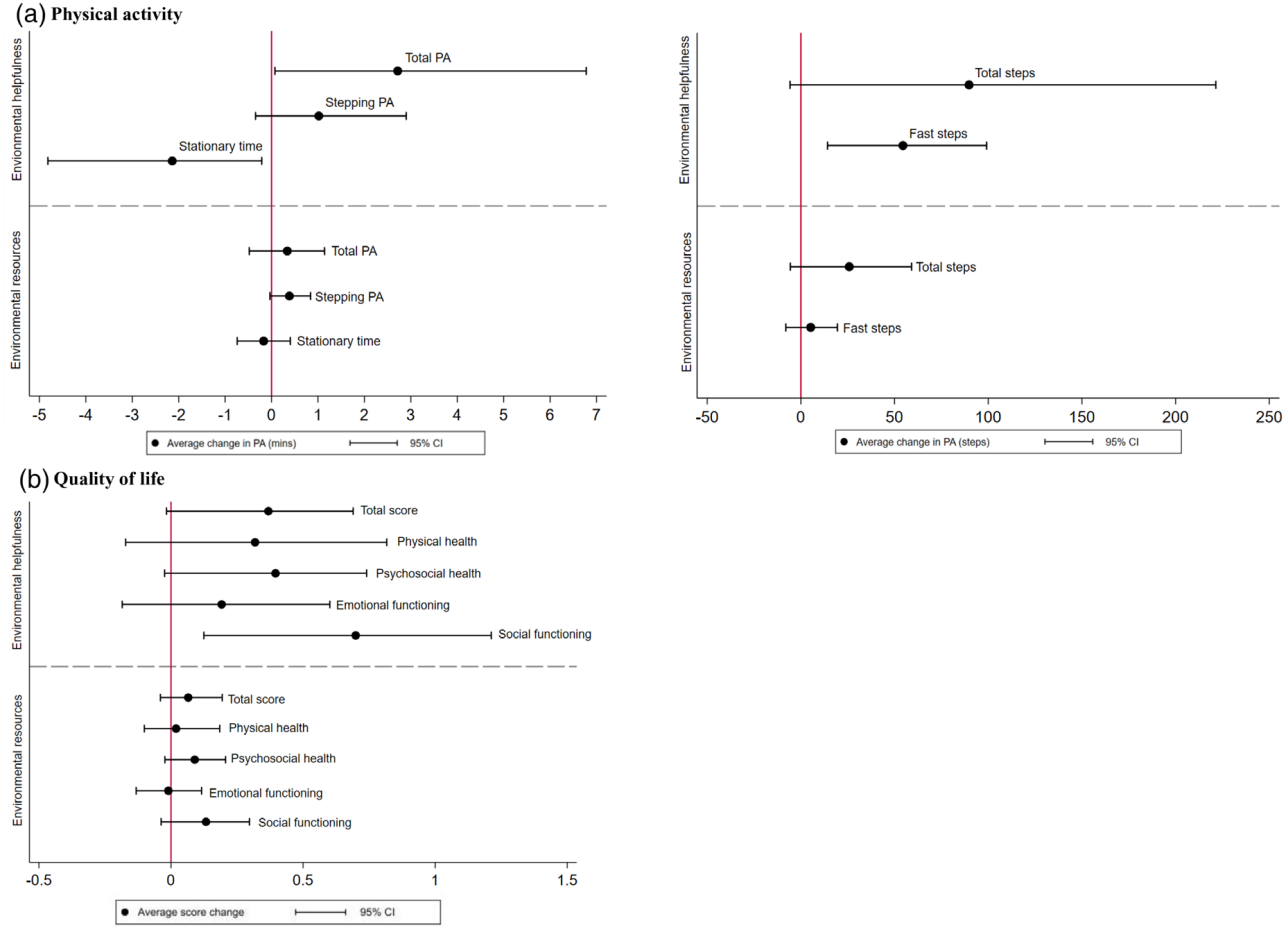
Estimated causal effect of environmental helpfulness and environmental resources on (a) physical activity and (b) quality of life. Abbreviations: CI, confidence interval; PA, physical activity.

Overall, estimates indicated that more helpful environmental features and greater availability of environmental resources were associated with more physical activity and less stationary time (Figure 1 and Table S3). In particular, associations were observed between environmental helpfulness and total physical activity, with each percentage‐point increase in environmental helpfulness score associated with an increase in mean total physical activity of 3 minutes (95% CI 0.1–7), and between environmental helpfulness and total steps, with each percentage‐point increase in environmental helpfulness associated with an increase in mean total steps of 90 steps (95% CI −6 to 99). The associations of environmental helpfulness and availability of environmental resources with total physical activity and stepping physical activity were slightly stronger for children born before 30 weeks compared with those born at term (Appendix S5); however, CIs were wide.

### Environment (helpfulness and resources) and QoL

Overall estimates also indicated that more helpful environmental features and greater availability of environmental resources were associated with a better QoL. The strongest associations were observed between environmental helpfulness and PedsQL social functioning score, with each increase in percentage point in environmental helpfulness associated with a social functioning score increase of 0.7 (95% CI 0.1–1.2) (Figure 1 and Table S3). The effect of environmental helpfulness and availability of environmental resources on QoL was similar between the birth groups (Table S4).

### Community participation (frequency and involvement) and physical activity

**FIGURE 2:**
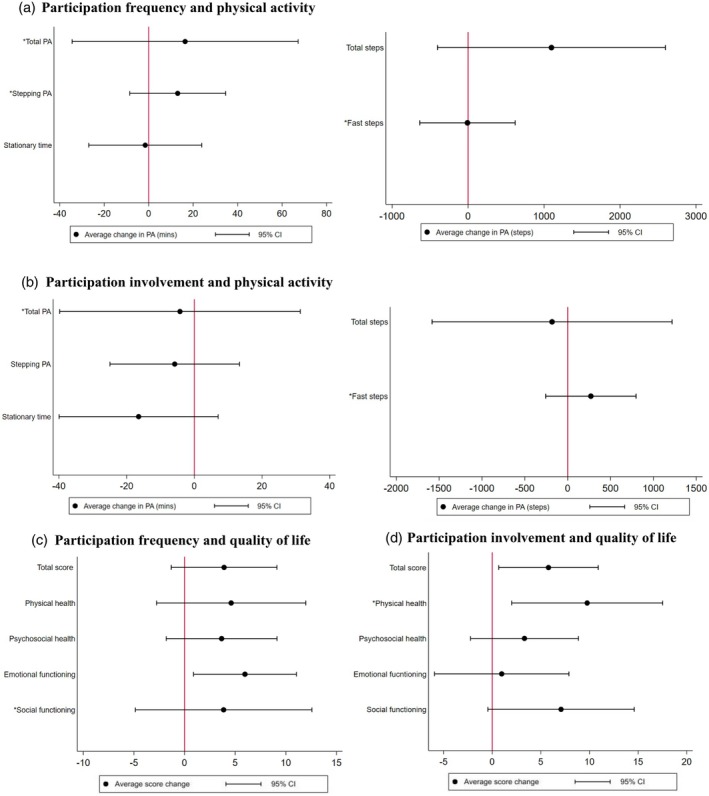
Associations between community participation with physical activity and quality of life. *Average change from linear regression models reported with robust standard errors as it was not possible to fit the model by generalized estimating equations owing to convergence issues. Abbreviations: CI, confidence interval; PA, physical activity.

Overall estimates indicated a positive association between frequency of community participation with higher physical activity and lower stationary time (Figure 2 and Table S3). For example, we found a mean difference of 1097 steps per day (95% CI −402 to 2597) for each point increase in participation frequency. We found weak evidence for a relationship between community participation frequency and involvement with all physical activity outcomes (Figure 2 and Table S3), and weak evidence that the association between community participation and physical activity differed between children born before 30 weeks and those born at term (Table S4).

### Community participation (frequency and involvement) and QoL

Overall estimates indicated a positive association between community participation and QoL, with stronger evidence for participation involvement than participation frequency (Figure 2 and Table S3). There was strong evidence that greater community participation involvement was associated with higher PedsQL total score (mean difference 5.8, 95% CI 0.7–10.9) and physical health summary score (mean difference 9.8, 95% CI 2.0–17.5) (Figure 2 and Table S3). There was also strong evidence that greater participation frequency was associated with higher emotional functioning score (mean difference 6.0, 95% CI 0.9–11.0). Overall, there was weak evidence that the association of community participation and QoL differed between children born before 30 weeks' gestation and those born at term (*p*‐values for interaction >0.05, Table S4).

## DISCUSSION

This study found that a more helpful and better resourced community environment could improve physical activity and QoL outcomes for 4‐ to 5‐year‐old children. Specifically, more helpful environmental features were associated with more total physical activity, more fast steps (more so for children born <30 weeks), less stationary time, and better social functioning. Better environmental resources have a smaller, although still positive, effect on physical activity and QoL. Under the assumptions of no confounding, no measurement error bias, and no selection bias (discussed further below), these estimated associations have a causal interpretation. These findings are in line with the assumptions in our directed acyclic graph. Indeed, improving aspects of environmental helpfulness, such as physical, social, and cognitive demands, safety of the community, and policies, are likely to improve the overall accessibility of an environment, which may explain the positive implications for physical activity and QoL. Likewise, better resources, such as access to adequate transport, services, time, and money are likely to have a similar, positive, impact on physical activity and QoL, which was evident in our findings, although the effect was weaker, which was unexpected. For our second aim, we found a positive relationship between higher frequency of participation and better emotional functioning as well as greater participation involvement and better overall QoL (PedsQL total score) and physical health.

Although there is no known minimally clinically important difference for accelerometer‐measured physical activity in children, greater involvement in community activities was associated with an improved QoL total score which exceeded the reported minimal clinically important difference for the PedsQL.^15^ This emphasizes the value of looking beyond attendance, to how involved and engaged children are in activities. Importantly, involvement is not a measure of activity competence, but instead is a subjective experience which can be different for each individual child.^5^

Physical activity has wide ranging benefits which are especially important for children born VPT, a population at higher risk of adverse health and developmental outcomes than their term‐born peers.^1^ Given that physical activity has a positive effect on physical (respiratory, cardiovascular, bone, and metabolic health) and mental health, and provides opportunities for children to develop motor skills while developing social connections and having fun, it is important to identify ways in which healthcare professionals can support children born VPT to be more active.^26^ Recent research has suggested that children born VPT are less active than their term‐born peers at preschool age.^14^, ^21^, ^27^ Furthermore, research indicates that subgroups of children born VPT, such as those with intraventricular haemorrhage or motor impairment, have poorer physical activity outcomes.^21^, ^27^ Such studies tend to focus on identifying which children are at risk of poor physical activity outcomes, which is necessary for recognizing those who need the most support to engage in physical activity. However, there are few studies exploring modifiable factors which have potential to be intervention targets. Aligned with our findings, results from a recent qualitative study exploring barriers and facilitators to physical activity participation for children born before 28 weeks' gestation emphasized the importance of environmental factors, such as social environment, coach characteristics, and recreational physical activity options, rather than child characteristics, when promoting physical activity for preschool‐age children.^7^

Similar to previous physical activity research, studies exploring QoL for children born VPT have largely focused on comparing QoL outcomes between children born VPT and term, with children born VPT having poorer QoL in the early years,^22^ and on predictors or risk factors for poor QoL. For example, for preschool‐age children born VPT, poorer QoL has been associated with lower gestational age, bronchopulmonary dysplasia^20^ and motor impairment.^28^ However, there is limited information on more modifiable factors, with potential to be intervention mechanisms for this population. Our findings that a more helpful environment could improve social functioning provides some small insight into this gap in the literature.

Although our study begins to address an important knowledge gap surrounding community participation for preschool‐aged children, we acknowledge several limitations. There are probably unmeasured confounders of the causal relationships between environmental helpfulness and resources, and QoL and physical activity that would lead to confounding bias. However, we have used directed acyclic graphs to clearly depict our causal assumptions and adjusted for key confounders. Our second aim examining the relationships between participation and physical activity or QoL was descriptive, and as such we encourage caution when interpreting our results. Further research, using longitudinal data, to explore the effects of participation (attendance and involvement) on physical activity and QoL is warranted. Another limitation is that we were missing YC‐PEM data for a part of the Victorian Infant Brain Study‐2 cohort. As outlined in Figure S2, the main reason for missing data was the decision not to approach families to complete the YC‐PEM (introduced part way through the data collection period) if they had already attended their 4‐ to 5‐year assessment, to minimize the burden on families.^6^ Furthermore, we had YC‐PEM data for more children born at term compared with VPT, which ultimately limited our precision to estimate differences between birth groups. When we introduced the YC‐PEM into the study, the proportion of children who had already completed the assessment happened to include a larger number in the VPT than the term group. This discrepancy was not noticed until data analysis, as all assessors were blinded to birth group. For our analysis we assumed that the individuals who had a completed YC‐PEM were representative of their birth group as a whole, and that no other factors caused missing data. While this is certainly a limitation, characteristics between children who did and did not have data for analysis in this study were largely similar (see Table S1). Further limitations to our study include the choice to use ankle‐mounted accelerometers, which cannot capture upper‐limb activity, and the possibility that the monitoring period was not representative of habitual physical activity for each child.

Of interest, our study found there was little difference in the effects of the community environment on physical activity and QoL when considering children born before 30 weeks and their term‐born peers separately. This may simply mean that the effect of community environmental helpfulness and resources are much the same for all children at preschool age. However, we acknowledge that our precision to estimate such effects is limited owing to our sample size. Furthermore, our small sample size prevented exploration of the effect of environmental features and helpfulness on physical activity and QoL for subgroups of children born before 30 weeks' gestation, such as those with motor impairment. Studies with larger sample sizes are warranted to better understand the effect of community participation on physical activity and QoL for children born VPT.

Our approach to accelerometery is a strength of this study, including our transparent reporting of accelerometer data management and processing protocols^14^ using raw acceleration recording, as physical activity research has been historically hampered by inconsistent data management and unclear reporting.^29^ Furthermore, our study used an individualized approach to measuring moderate to vigorous physical activity (fast steps) and used several accelerometer‐measured outcomes of physical activity. A further strength of this study is the use of a participation outcome that measures both attendance and involvement, and considers contextual environmental factors.^5^

This paper extends our understanding of potential benefits of community participation for preschool‐age children born VPT; however, our findings should be interpreted with caution considering our sample size and other limitations. Overall, this study adds to the expanding body of participation literature, highlighting that context and environment influence physical activity and QoL for children.^5^, ^30^ Encouragingly, it is likely that strategies to improve environmental helpfulness (such as adapting physical, social, or sensory aspects of community activities) may have positive effects on physical activity and QoL outcomes for all children, while recognizing that children born VPT may experience greater barriers to participation.^6^ For example, environmental features commonly reported as barriers included the physical layout, and the physical and social demands of community activities (Table S2 and Figure S3), many of which are modifiable. This is in line with contemporary understanding of childhood disability, which moves away from impairment‐focused interventions and towards participation, goal setting, and inclusion.^30^ For example, there is evidence that adapting environmental factors, such as providing educational materials or support for sports coaches, adjusting task requirements, or providing supportive social environments to try new activities, improves physical activity outcomes for children with disabilities.^31^, ^32^ For clinicians, this reinforces the need to consider intervention approaches that focus on modifying the task or environment when working towards physical activity or QoL goals. Importantly, components of a child's environmental resources, such as access to public transport, availability of suitable activities, and financial support, are not aspects of participation that are suitable, or even possible, for health professionals to address alone.^30^ However, the impact of social, political, and physical environments on each individual child's health should be considered when working with families. Further research is needed to understand how to better promote physical activity and QoL for children born VPT, with a focus on modifiable correlates that could contribute to an understanding of how to best structure interventions. Interventions should be designed with children and families at their centre and include methodologies that recognize children and families as experts in their own health and participation.

## CONFLICT OF INTEREST STATEMENT

The authors have stated that they had no interests that might be perceived as posing a conflict or bias.

## Supporting information


**Table S1:** Participant characteristics of children with data for analysis (YC‐PEM data plus accelerometer and/or PedsQL data) versus those without.

**Table S2:** Participation frequency and involvement for each community activity item presented separately for children born very preterm and at term.

**Table S3:** Mean difference in physical activity and QoL variables per one‐unit increase in participation frequency/involvement score and environmental helpfulness/resources percentage point.

**Table S4:** Mean difference in physical activity and QoL variables per one‐unit increase in participation frequency/involvement score and environmental helpfulness/resources percentage point by birth group.

**Figure S1:** Directed acyclic graph for outcomes of interest at 4 to 5 years corrected age depicting assumed causal relationship between variables.

**Figure S2:** Flow of participants through the study.

**Figure S3:** Perceived helpfulness of environmental factors to community participation: parents of children born <30 weeks and at term.

## Data Availability

Data available on request from the authors.
