# Supplementary material for: Community participation, physical activity, and quality of life for children born very preterm
Source: Dev Med Child Neurol. 2025 Mar 20;67(10):1331–9. doi: 10.1111/dmcn.16295 (PMC12426303; doi:10.1111/dmcn.16295)
Supplement: Supplementary file 1 — Table S1: Participant characteristics of children with data for analysis (YC‐PEM data plus accelerometer and/or PedsQL data) versus those without. [file DMCN-67-1331-s001.docx]

Table S1. Participant characteristics of children with data for analysis (YC-PEM data plus accelerometer and/or PedsQL data) vs. those without

|  | Born <30 weeks’ gestation | | Born at term | |
| --- | --- | --- | --- | --- |
|  | Participants (n=45) | Non-participants (n=78) | Participants (n=89) | Non-participants (n=39) |
| Gestational age (weeks), mean (SD) | 27.7 (1.7) | 27.9 (1.4) | 39.9 (1.2) | 39.8 (1.1) |
| Multiple births, n (%) | 20 (44) | 31 (40) | 2 (2) | 0 (0) |
| Sex (male), n (%) | 21 (47) | 39 (50) | 44 (49) | 18 (46) |
| Higher social risk, n (%) | 18/39 (46)^*^ | 25/65 (38)^*^ | 22/85 (26)^*^ | 6/31 (19)^*^ |
| CA at assessment (years), mean (SD) | 4.7 (0.1) | 4.7 (0.1) | 4.8 (0.2) | 4.9 (0.2) |
| MABC-2 ≤16th centile and/or cerebral palsy, n (%) | 17/44 (39)^*^ | 23/77 (30)^*^ | 12/87 (13)^*^ | 7/37 (19)^*^ |
| Cerebral palsy diagnosis, n (%) | 3 (7) | 3 (4) | 0 (0) | 0 (0) |
| L-DCDQ suspect for DCD, n (%) | 21/41 (51)^*^ | 30/66 (45)^*^ | 29/83 (35)^*^ | 10/28 (36)^*^ |
| Autism spectrum disorder diagnosis, n (%) | 2 (4) | 6 (8) | 2 (2) | 0 (0) |

^*^ n/number with available data, n; number, SD; standard deviation, CA; corrected age, MABC-2, Movement Assessment Battery for Children, 2^nd^ edition; L-DCDQ, Little Developmental Coordination Disorder Questionnaire; DCD, Developmental Coordination Disorder.
