# Supplementary material for: Community participation, physical activity, and quality of life for children born very preterm
Source: Dev Med Child Neurol. 2025 Mar 20;67(10):1331–9. doi: 10.1111/dmcn.16295 (PMC12426303; doi:10.1111/dmcn.16295)
Supplement: Supplementary file 2 — Table S2: Participation frequency and involvement for each community activity item presented separately for children born very preterm and at term. [file DMCN-67-1331-s005.docx]

Table S2: Participation frequency and involvement for each community activity item presented separately for children born very preterm and at term.

| **Children born <30 weeks n=45** | | | | **Children born term n=89** | | |
| --- | --- | --- | --- | --- | --- | --- |
|  | **Never participates** | **Frequency** | **Involvement *** | **Never participates** | **Frequency** | **Involvement*** |
| **Participation Item** | n (%) | mean (SD)  range | mean (SD)  range | n (%) | mean (SD)  range | mean (SD)  range |
| Shopping and errands | 0 (0%) | 5.4 (1.2)  2-7 | 4.1 (0.9)  3-5 | 1 (1%) | 5.7 (1.0)  0-7 | 4.3 (1.0)  1-5 |
| Dining out | 2 (4%) | 4.3 (1.6)  0-6 | 4.3 (0.9)  3-5 | 0 (0%) | 4.7 (1.2)  1-7 | 4.6 (0.8)  3-5 |
| Routine appointments | 0 (0%) | 3.3 (1.2)  1-5 | 4.1 (1.0)  1-5 | 1 (1%) | 3.1 (1.0)  0-6 | 4.1 (1.0)  1-5 |
| Classes and lessons | 7 (16%) | 4.1 (2.3)  0-7 | 4.3 (1.0)  2-5 | 30 (34%) | 3.4 (2.7)  0-7 | 4.7 (0.8)  1-5 |
| Organised physical activity | 7 (16%) | 3.9 (2.1)  0-7 | 4.6 (0.8)  3-5 | 11 (12%) | 4.5 (2.0)  0-7 | 4.6 (0.7)  3-5 |
| Community attractions | 0 (0%) | 3.3 (1.4)  1-6 | 4.5 (1.0)  1-5 | 1 (1%) | 4.1 (1.4)  0-6 | 4.6 (0.7)  3-5 |
| Religious or spiritual gatherings | 29 (64%) | 0.8 (1.4)  0-5 | 3.3 (1.4)  1-5 | 72 (81%) | 0.5 (1.3)  0-6 | 3.2 (1.6)  1-5 |
| Social gatherings | 0 (0%) | 3.7 (1.5)  1-7 | 4.5 (0.8)  1-5 | 1 (1%) | 3.9 (1.3)  0-7 | 4.6 (0.8)  3-5 |
| Community events | 2 (4%) | 2.8 (1.5)  0-7 | 4.3 (0.8)  3-5 | 3 (3%) | 2.7 (1.2)  0-5 | 4.35 (1.0)  1-5 |
| Unstructured physical activities | 0 (0%) | 5.4 (1.3)  1-7 | 4.8 (0.8)  1-5 | 0 (0%) | 5.9 (1.0)  1-7 | 4.8 (0.6)  2-5 |
| Overnight trips/vacations | 1 (2%) | 2.2 (1.4)  0-5 | 4.5 (1.2)  1-5 | 5 (6%) | 2.6 (1.3)  0-5 | 4.7 (0.9)  1-5 |

*of children that participated in the activity. n=number, SD=standard deviation, VPT=very preterm
