# Supplementary material for: Community participation, physical activity, and quality of life for children born very preterm
Source: Dev Med Child Neurol. 2025 Mar 20;67(10):1331–9. doi: 10.1111/dmcn.16295 (PMC12426303; doi:10.1111/dmcn.16295)
Supplement: Supplementary file 4 — Table S4: Mean difference in physical activity and QoL variables per one‐unit increase in participation frequency/involvement score and environmental helpfulness/resources percentage point by birth group. [file DMCN-67-1331-s006.docx]

**Table S4**

Mean difference (MD) in PA and QoL variables per one-unit increase in participation frequency/involvement score by birth group

| **Physical activity** | | | | | | | | | | | | | | | | | | | | | | | | | | | | | | | | | | | | | | | | | | |
| --- | --- | --- | --- | --- | --- | --- | --- | --- | --- | --- | --- | --- | --- | --- | --- | --- | --- | --- | --- | --- | --- | --- | --- | --- | --- | --- | --- | --- | --- | --- | --- | --- | --- | --- | --- | --- | --- | --- | --- | --- | --- | --- |
| MD (95% CI) p values | | | | | | | | | | | | | | | | | | | | | | | | | | | | | | | | | | | | | | | | | | |
| **YC-PEM variables** | **^*^Total PA (mins/day)** | | | | | | | | | **Stepping time (mins/day)** | | | | | | | | **Stationary time (mins/day)** | | | | | | | | **Steps/day (n)** | | | | | | | | | **^*^Fast step/day (n)** | | | | | | | |
|  | **<30 weeks** | | **Term-born** | | | | **<30 weeks** | | | | | **Term-born** | | | | **<30 weeks** | | | | | | **Term-born** | | | | **<30 weeks** | | | | **Term-born** | | | | | **<30 weeks** | | | | **Term-born** | | | |
| Frequency | 7 (-105, 118); p=0.91 | | 15 (-36, 65); p=0.57 | | | | 3 (-35, 40); p=0.89 | | | | | 17 (-9, 43); p=0.21 | | | | -4 (-43, 35); p=0.83 | | | | | | 2 (-31,36); p=0.89 | | | | 543 (-1868, 2954); p=0.66 | | | | 1172 (-745, 3090); p=0.23 | | | | | 118 (-1063, 1298); p=0.84 | | | | 2 (-737, 741); p=0.99 | | | |
| Quality | -31 (-91, 29); p=0.31 | | 8 (-34, 51); p=0.69 | | | | -10 (-33, 14); p=0.42 | | | | | -3 (-30, 24); p=0.81 | | | | -17 (-45, 10); p=0.22 | | | | | | -15 (-48, 17); p=0.35 | | | | -385 (-1935, 1164); p=0.62 | | | | -65 (-2076, 194); p=0.95 | | | | | 485 (-478, 1447); p=0.32 | | | | 170 (-468, 809); p=0.60 | | | |
| **Quality of life** | | | | | | | | | | | | | | | | | | | | | | | | | | | | | | | | | | | | | | | | | | |
| MD (95% CI) p values | | | | | | | | | | | | | | | | | | | | | | | | | | | | | | | | | | | | | | | | | | |
| **YC-PEM variables** | **Total score** | | | | | | | | | | **Physical health** | | | | | | | | | | **Psychosocial health** | | | | | **Emotional functioning** | | | | | | | | **Social functioning** | | | | | | | | |
|  | **<30 weeks** | **Term-born** | | | | **<30 weeks** | | | | | | | **Term-born** | | | | **<30 weeks** | | | | | | **Term-born** | | | | **<30 weeks** | | | | **Term-born** | | | | | **<30 weeks** | | | | **Term-born** | | |
| Frequency | **^*^**-0.6 (-11.1, 9.9); p=0.91 | | | **^*^**4.5 (-2.7, 11.7); p=0.21) | | | | -5.5 (-15.8, 4.8); p=0.30 | | | | | | 7.1 (-3.5, 17.7); p=0.19 | | | | | -1.4 (-9.1, 11.9); p=0.79 | | | | | 3.2 (-3.8, 10.1); p=0.37 | | | | 8.1 (-0.7, 17.0); p=0.07 | | | | 4.1 (-2.0, 10.3); p=0.19) | | | | | **^*^**1.3 (-18.4, 21.0); p=0.90 | | | | **^*^**4.0 (-5.4, 13.5); p=0.40 | |
| Quality | **^*^**3.9 (-4.6, 12.4); p=0.36 | | | | **^*^**6.3 (-0.9, 13.5); p=0.08 | | | | **^*^**7.3 (-4.8, 19.5); p=0.23 | | | | | | **^*^**10.8 (-0.7, 21.0); p=0.037 | | | | | 0.8 (-8.1, 9.7); p=0.87 | | | | | 3.9 (-3.1, 11.0);p=0.28 | | | | **^*^**1.9 (-13.8, 17.6); p=0.81 | | | | **^*^**1.0 (-7.0, 9.0); p=0.81 | | | | | **^*^**6.0 (-9.3, 21.4); p=0.44 | | | | **^*^**7.0 (-2.5, 16.4); p=0.15 |

Estimates are from univariable linear regression models fitted using generalised estimating equations (GEEs) to allow for clustering among multiple births within a family and using robust (sandwich) standard errors. *Models fitted with robust standard errors as it was not possible to fit the model with GEEs due to convergence issues. CI= confidence interval; PA=physical activity; QoL= Quality of Life, YC-PEM= Young Children’s Participation & Environment Measure

Mean difference (MD) in PA and QoL variables per one-unit increase in environmental helpfulness/resources percentage point by birth group

| **Physical activity** | | | | | | | | | | |
| --- | --- | --- | --- | --- | --- | --- | --- | --- | --- | --- |
| MD (95% CI) | | | | | | | | | | |
| **YC-PEM variables** | **Total PA (mins/day)** | | **Stepping time (mins/day)** | | **Stationary time (mins/day)** | | **Steps/day (n)** | | **Fast step/day (n)** | |
|  | **<30 weeks** | **Term-born** | **<30 weeks** | **Term-born** | **<30 weeks** | **Term-born** | **<30 weeks** | **Term-born** | **<30 weeks** | **Term-born** |
| Environmental helpfulness | 4.2 (-0.3, 8.9) | 2.1 (-1.4, 7.2) | 1.4 (-0.8, 3.4) | 0.9 (-0.8, 3.4) | -2.4 (-4.9, -0.06) | -2.0 (-5.9, 0.3) | 110 (-39, 255) | 82 (-38, 271) | 103 (12, 197) | 35 (-12, 83) |
| Environmental resources | 1.0 (-1.0, 2.7) | 0.09 (-0.8, 0.9) | 0.5 (-0.3, 1.1) | 0.3 (-0.2, 0.9) | -0.6 (-1.4, 0.5) | -.01 (-0.8, 0.7) | 33 (-18, 76) | 23 (-17, 65) | 7 (-27, 48) | 4 (-10, 17) |
| **Quality of life** | | | | | | | | | | |
| MD (95% CI) | | | | | | | | | | |
| **YC-PEM variables** | **Total score** | | **Physical health** | | **Psychosocial health** | | **Emotional functioning** | | **Social functioning** | |
|  | **<30 weeks** | **Term-born** | **<30 weeks** | **Term-born** | **<30 weeks** | **Term-born** | **<30 weeks** | **Term-born** | **<30 weeks** | **Term-born** |
| Environmental helpfulness | 0.5 (-0.2, 1.1) | 0.3 (-0.1, 0.7) | 0.7 (-0.3, 1.5) | 0.2 (-0.4, 0.8) | 0.5 (-0.3, 1.0) | 0.4 (-0.2, 0.8) | 0.1 (-0.5, 0.7) | 0.2 (-0.2, 0.7) | 0.9 (-0.3, 1.9) | 0.6 (-0.05, 1.2) |
| Environmental resources | 0.09 (-0.1, 0.4) | 0.05 (-0.06, 0.16) | 0.08 (-0.3, 0.5) | -0.004 (-0.1, 0.1) | 0.1 (-0.1, 0.4) | 0.08 (-0.06, 0.2) | 0.2 (-0.001, 0.4) | -0.09 (-0.2, 0.05) | 0.08 (-0.3, 0.5) | 0.1 (-0.03, 0.3) |

Estimates are from g-computation. Linear regression models were used to predict the outcome under different exposure values. Models included adjustment for social risk status, birth group and motor impairment (models involving QoL only). Confidence intervals obtained using a non-parametric cluster bootstrap (percentile method) to account for clustering among multiple births within a family. CI= confidence interval; QoL= Quality of Life; YC-PEM= Young Children’s Participation & Environment Measure
