# Supplementary material for: Community participation, physical activity, and quality of life for children born very preterm
Source: Dev Med Child Neurol. 2025 Mar 20;67(10):1331–9. doi: 10.1111/dmcn.16295 (PMC12426303; doi:10.1111/dmcn.16295)
Supplement: Supplementary file 5 — Figure S1: Directed acyclic graph for outcomes of interest at 4 to 5 years corrected age depicting assumed causal relationship between variables. [file DMCN-67-1331-s002.docx]

Figure S1: Directed acyclic graph for outcomes of interest at 4-5 years’ corrected age depicting assumed causal relationship between variables.

Birth group

(very preterm vs term)

Social risk status (higher vs. lower)

**Exposures:**

Helpful environmental features

Availability of environmental resources

**Outcome:**

Physical activity

**Exposures:**

Helpful environmental features

Availability of environmental resources

**Outcome:**

Quality of Life

Motor impairment

Social risk status (higher vs. lower)

Birth group

(very preterm vs term)
