# Supplementary figures and images for: Community participation, physical activity, and quality of life for children born very preterm
Source: Dev Med Child Neurol. 2025 Mar 20;67(10):1331–9. doi: 10.1111/dmcn.16295 (PMC12426303; doi:10.1111/dmcn.16295)

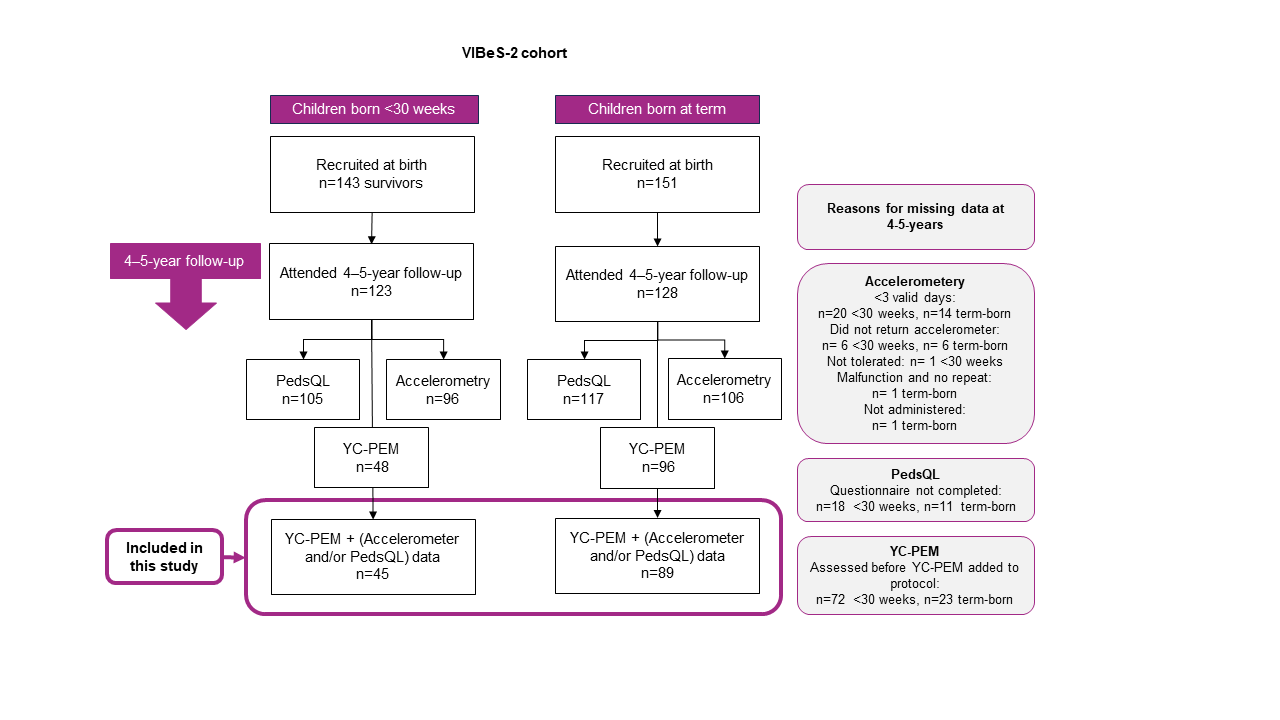

Supplement: Supplementary file 6 — Figure S2: Flow of participants through the study. [file DMCN-67-1331-s007.png]
