# Supplementary material for: Community participation, physical activity, and quality of life for children born very preterm
Source: Dev Med Child Neurol. 2025 Mar 20;67(10):1331–9. doi: 10.1111/dmcn.16295 (PMC12426303; doi:10.1111/dmcn.16295)
Supplement: Supplementary file 7 — Figure S3: Perceived helpfulness of environmental factors to community participation: parents of children born <30 weeks and at term. [file DMCN-67-1331-s003.docx]

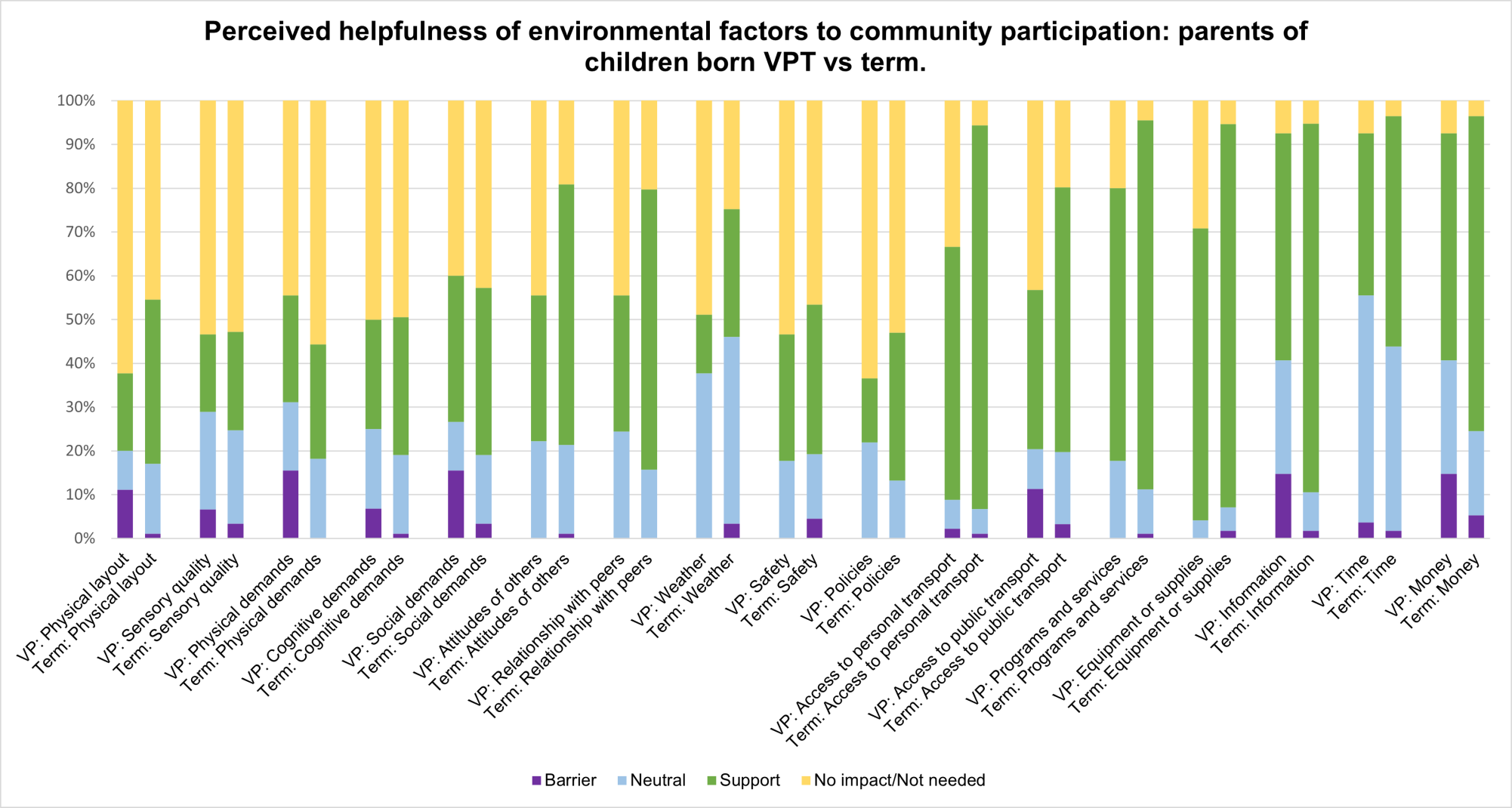


Figure S3: Perceived helpfulness of environmental factors to community participation: parents of children born <30 weeks and at term. Percentage of parents who indicated an environmental factor or resource was a barrier (usually makes harder, or usually no), neutral (sometimes helps, sometimes makes harder or sometimes yes, sometimes no) a support (usually helps, usually yes) or no impact/not needed.
